# Enoxaparin-induced reactive thrombocytosis: a case report

**DOI:** 10.1186/s12959-021-00290-x

**Published:** 2021-05-26

**Authors:** Tao Xiang, Ming Cheng

**Affiliations:** Department of Rehabilitation, Chengdu Jinniu District People’s Hospital, 300 Jinfu Road, Jinniu District, Chengdu City, 610000 Sichuan Province China

**Keywords:** Enoxaparin, Thrombocytosis, Adverse drug reaction

## Abstract

**Background:**

Enoxaparin is an anticoagulant that falls in the class of medications called low molecular weight heparins (LMWHs), and is used to prevent or treat patients with deep vein thrombosis (DVT) and pulmonary embolism. Enoxaparin is the most widely used LMWH for DVT prophylaxis following knee or hip replacement surgery. Common side effects of enoxaparin include bleeding, petechiae at the injection site, and thrombocytopenia. However, reactive thrombocytosis is a rarely reported adverse reaction. We managed a patient who developed enoxaparin-associated thrombocytosis, which was completely resolved after treatment cessation.

**Case presentation:**

A 78-year-old female was hospitalized for post-hip replacement rehabilitation. Low molecular weight heparin 40 mg/day was administered subcutaneously to prevent deep venous thrombosis (DVT). At admission, her platelet count was normal (228 × 10^9^/L) and her white blood cell count was slightly elevated (12.91 × 10^9^/L). Seven days after admission, the patient developed thrombocytosis, which peaked on the 14th day (836 × 10^9^/L), while her white blood cell count had returned to normal (8.86 × 10^9^/L). Her therapeutic regimen was reviewed, and enoxaparin was identified as a potentially reversible cause of reactive thrombocytosis. Switching from enoxaparin to rivaroxaban lead to a gradual decrease in the patient’s platelet count, which eventually returned to normal levels 16 days after enoxaparin was discontinued. No complications secondary to thrombocytosis was observed, and no conclusion was reached on the use of small doses of aspirin for antithrombotic therapy under these circumstances.

**Conclusion:**

Enoxaparin-induced reactive thrombocytosis should be suspected in patients with thrombocytosis following enoxaparin administration as an anticoagulant to prevent certain complications.

## Background

Enoxaparin is a low molecular weight heparin (LMWH) used to prevent or treat deep vein thrombosis (DVT) and pulmonary embolism [1]. It generally induces a factor Xa to factor IIa ratio greater than 4, eliciting a strong antithrombotic effect that dissolves thrombi. Compared to standard unfractionated heparin (UFH), the bioavailability of LMWHs is higher after i.v or s.c administration, and was estimated to range from 90 to 95%; the biological half-life (Based on anti-Xa clearance) of LMWHs is twice longer than that of UFH. The t1/2 of LMWHs has been reported to be 100 min after i.v administration and 360 min after s.c administration, respectively [2]. Enoxaparin is the most widely used LMWH for DVT prophylaxis following knee or hip replacement surgery [3]. The most common side effects of enoxaparin include bleeding, petechiae at the injection site, and thrombocytopenia. Nonetheless, some rare cases of enoxaparin-induced reactive thrombocytosis have been reported. We treated a patient who developed enoxaparin-associated thrombocytosis, which resulted in complete resolution after enoxaparin was discontinued.

## Case presentation

A 78-year-old Asian female was admitted to the rehabilitation department roughly 8 h after receiving a hip replacement surgery. Her medical history was significant for hypertension and atrial fibrillation. At admission, her baseline platelet count was 228 × 10^9^/L (Normal range 85–303 × 10^9^/L), and her white blood cell count was 12.91 × 10^9^/L (Normal range 3.5–9.2 × 10^9^/L). After the risk assessment for venous thrombus embolism (VTE) was performed, a prophylactic regimen for deep vein thrombosis (DVT) consisting of subcutaneous enoxaparin 4000 IU/day was initiated.

Seven days after admission (Hospital day 7), the patient’s platelet count rose to 494 × 10^9^/L, while the white blood cell count fell to 10.21 × 10^9^/L. The patient had no symptoms or signs of thrombosis, such as swelling or pain in her lower extremities. Thus, we suspected a reactive thrombocytosis due to post-surgical inflammation. On the 10th day of hospitalization, her laboratory findings delineated a raised platelet count (739 × 10^9^/L) and a normal white blood cell count (9.18 × 10^9^/L). The patient’s platelet count kept rising steadily; after 14 days of subcutaneous enoxaparin administration, the patient’s platelet count peaked at 836 × 10^9^/L (Hospital day 14), and her white blood cell count was still within the normal range (8.86 × 10^9^/L). At this point, the changes observed in this patient’s platelet count were no longer consistent with postoperative inflammation, hence we began to consider other etiologic factors for this patient’s reactive thrombocytosis. The patient’s therapeutic regimen was reviewed and enoxaparin was identified as the only potential pharmacologic cause of thrombocytosis. Enoxaparin was immediately discontinued. Previous studies did not find similar adverse reactions to rivaroxaban, so starting from hospital day 16, enoxaprin was replaced by rivaroxaban 15 mg daily.

**Fig. 1:**
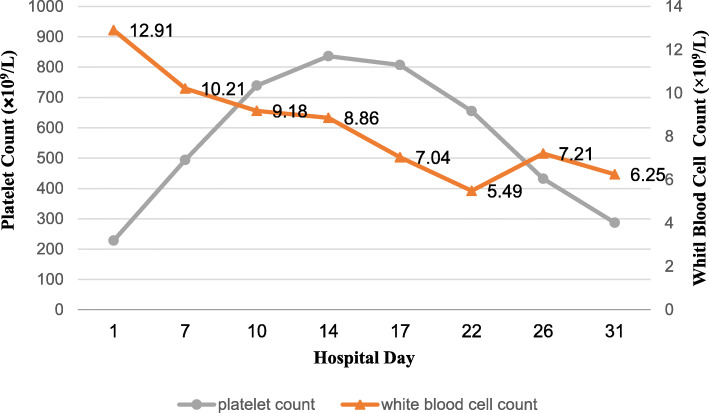
Timeline of the patient’s hospital course indicating platelet and white blood cell counts

A decline in the patient’s platelet count was observed the next morning (Hospital day 17). The patient’s platelet count eventually began to decrease gradually, reaching 655 × 10^9^/L 5 days after enoxaparin was discontinued (hospital day 22). Finally, after 15 days of enoxaparin discontinuation, the patient had a normal platelet count of 286 × 10^9^/L (hospital day 31). Figure 1 illustrates a timeline of the patient’s hospital course, platelet count, and white blood cell count.

The patient’s hemoglobin count, mean erythrocyte volume, mean erythrocyte hemoglobin concentration, procalcitonin and C-reactive protein levels were normal throughout her hospital stay, and her serum iron levels were not monitored.

The patient was discharged 32 days after admission with no bleeding or thrombotic manifestations. On follow-up, her platelet count was still within the normal range.

## Discussion

According to the baseline essential thrombocytosis threshold determined by the World Health Organization [4], a platelet count exceeding 450 × 10^9^ cells/L is defined as thrombocytosis. Thrombocytosis is classified as either primary (Essential) or secondary (Reactive). The most common etiologies for reactive thrombocytosis are inflammation, tissue damage, infection, hyposplenism, iron deficiency, haemolysis, drug reactions, and other factors inducing an acute phase response [5]. In our patient, the potential causes of thrombocytosis were surgery, infection, and drug reactions.

This patient had a normal platelet count and an increased white blood cell count on hospital admission. During her first 14 days in the hospital, her white blood cell count gradually returned to normal, while her platelet count kept spiking and eventually peaking at 836 × 10^9^/L. Meanwhile, her procalcitonin and C-reactive protein levels remained normal. Therefore, the patient’s laboratory values were neither consistent with inflammation nor infection as contributing factors to her increased platelet count. Anemia was not supported based on her laboratory indicators and clinical symptoms. We also excluded bleeding as a possible cause for the patient’s thrombocytosis, so drug-induced reactive thrombocytosis was the only potential culprit left.

In addition to the subcutaneous injections of enoxaparin, the patient was given oral medications including amlodipine, telmisartan, aspirin, and atorvastatin. Previous research have revealed that some medications including All-Trans Retinoic Acid (ATRA), Antibiotics, Clozapine, Epinephrine, Gemcitabine, Low-Molecular-Weight Heparins (LMWHs), and Vinca Alkaloids may lead to drug-induced thrombocytosis, and the strongest evidence of causality supports LMWH and neonatal drug withdrawal as the main etiologic factors [6]. Based on this facts, we suspected that the patient’s thrombocytosis was induced by enoxaparin, so anticoagulation with rivaroxaban instead was initiated on the 16th day of hospitalization. Subsequently, a gradual decrease in the patient’s platelet count was observed from the second day following enoxaparin discontinuation, which eventually returned to normal after 16 days of enoxaparin cessation. Thus, the temporal correlation between enoxaparin and thrombocytosis was clearly demonstrated. To further verify our hypothesis and confirm the possibility of drug-induced thrombocytosis, the Modified Naranjo Scale with Thrombocytosis-Specific Criteria [6] was used. Our patient’s score was 7, so we concluded that the reactive thrombocytosis was most likely associated with enoxaparin.

Furthermore, previous reports of LMWH causing reactive thrombocytosis have been published. Through review of the literature, we found that patients developed thrombocytosis 14 days on average (Range = 3–35 days) after LMWH administration, which is consistent with the findings observed in our case report. The mechanism by which LMWH exposure leads to thrombocytosis has not yet been elucidated [7]. In a mouse experiment, an increase in murine platelet counts, immature megakaryocytes, and colony-forming unit megakaryocytes in the bone marrow after intraperitoneal administration of 0.1–25 IU of fraxiparin for 4 days was observed. The author emitted the hypothesis that fraxiparin acted synergistically with heparin cofactor II and antithrombin III to promote megakaryocyte colony formation [8]. Another report showed that heparin significantly potentiates the megakaryocytopoietic activity of the C-Mpl ligand and interleukin (1 L)-6, but not that of IL3, GM-CSF, SCF, and Epo. Additionally, heparin was found to significantly neutralize the inhibitory actions of platelet factor 4 (PF4) and transforming growth factor β1 (TGFβ1) on megakaryocyte colony growth [9]. We hypothesized that LMWH may stimulate hematopoiesis in the bone marrow, leading to an increase in platelets.

## Conclusions

In conclusion, enoxaparin-induced reactive thrombocytosis should be suspected in patients with thrombocytosis following enoxaparin administration in order to prevent further complications. It is generally believed that reactive thrombocytosis does not represent a relevant thrombotic risk [10], and patients rarely experience symptoms or any clinical manifestation from reactive thrombocytosis [11, 12]. Moreover, no conclusion was reached on the use of low-dose aspirin as an antithrombotic agent to prevent thrombosis in this kind of patients. Thus, clinicians should evaluate the thrombotic and bleeding risks on a case-by-case basis to come up with the most appropriate management plan for each patient.

## Data Availability

The datasets obtained and analyzed in the current study are available from the corresponding author on reasonable request.
